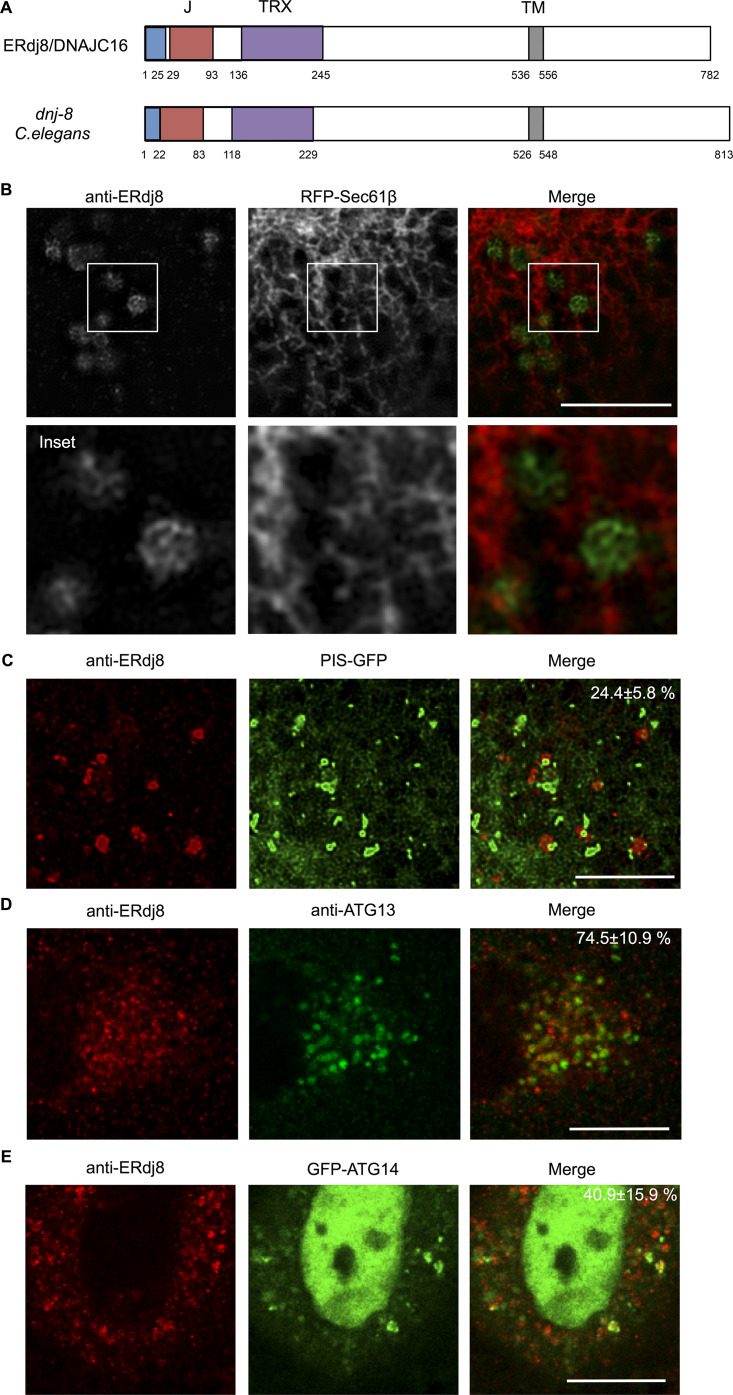# Correction: ERdj8 governs the size of autophagosomes during the formation process

**DOI:** 10.1083/jcb.20190312709142020c

**Published:** 2020-09-23

**Authors:** Yo-hei Yamamoto, Ayano Kasai, Hiroko Omori, Tomoe Takino, Munechika Sugihara, Tetsuo Umemoto, Maho Hamasaki, Tomohisa Hatta, Tohru Natsume, Richard I. Morimoto, Ritsuko Arai, Satoshi Waguri, Miyuki Sato, Ken Sato, Shoshana Bar-Nun, Tamotsu Yoshimori, Takeshi Noda, Kazuhiro Nagata

Vol. 219, No. 8 | 10.1083/jcb.201903127 | June 3, 2020

The authors have noticed an error in Figure 1 D of the original publication. The authors inadvertently reversed the labeling of for the anti-ERdj8 and the anti-ATG13 signals. Consequently, the labeling of the merged image was also reversed. The corrected figure is shown below. This change does not alter the interpretation of the figure or the conclusions of the manuscript. This error appears only in PDF versions downloaded on or before September 23, 2020.

**Figure:**